# Perspective: Milk and Dairy Provide Affordable High-Quality Protein and Merit Inclusion in the Protein Foods Group

**DOI:** 10.1016/j.cdnut.2024.104539

**Published:** 2024-12-21

**Authors:** Adam Drewnowski

**Affiliations:** Center for Public Health Nutrition, University of Washington, Seattle, WA, United States

**Keywords:** USDA protein foods group, dairy group, 1-ounce protein equivalents, Protein Digestibility Corrected Amino Acid Score (PDCAAS), nutrient density, Nutrient Rich Foods Index (NRF), national food prices, affordability

## Abstract

The United States Department of Agriculture (USDA) protein foods group includes meat, poultry, seafood, and several plant-based proteins—but not dairy. Yet milk, yogurt, and cheese provide affordable high-quality protein and multiple priority micronutrients. The present analyses compared dairy with Protein Group foods in terms of protein content and quality, nutrient density, and protein cost, using USDA nutrient composition databases and published national food prices. Protein quality was adjusted using Protein Digestibility Corrected Amino Acid Scores (PDCAAS). Nutrient density was assessed using the Nutrient Rich Food Index calculated per 100 kcal and per serving. Affordability was the cost of providing 1-oz equivalent of PDCAAS-corrected high-quality protein. Servings of milk, yogurt, and cheese provided 7 g of protein and were comparable with eggs and beans in terms of protein cost. The MyPlate 1-oz protein equivalency table ought to consider protein quality and ought to include dairy products as sources of dietary protein.

## Introduction

The recommended daily amount of protein for healthy adults is between 10% and 35% of total energy needs [1]. To promote healthy diet choices, the USDA [2] recommends a wide variety of protein foods, both animal and plant. Foods in the USDA protein foods group include all foods made from seafood, meat, poultry, and eggs, along with pulses, nuts, seeds, and soy products [2,3].

Placing foods in the USDA protein foods group can be viewed as an implicit protein content claim. The term “1-oz equivalent of protein” specifically refers to the amounts of food from the protein foods group that provide roughly 7 g of protein [2]. The USDA MyPlate guidelines [2] list “equivalent” protein amounts as 1 oz of meat, poultry, and fish, ¼ cup of cooked beans or tofu, 1 egg, 1 tbs of peanut butter (14 g), and ½ oz of nuts or seeds [2].

The USDA protein foods group does not include milk or dairy products [2]. Yet milk and dairy are significant sources of affordable high-quality protein and contain multiple bone-building micronutrients [[4], [5], [6]]. Pulses (beans, peas, and lentils) are listed as vegetables and as sources of plant protein. Dairy could retain its status as a distinct group (and major source of calcium and vitamin D) and also be listed among protein foods of choice [3].

The “equivalency” of proteins from various sources is revisited in this perspective. First, not all foods in the protein foods group contain equal amounts of protein. Mean protein content of foods (in grams of protein per 100 g of food) can range from 6 g/100 g for pulses to ≤26 g/100 g for pork, beef, and lamb [7]. Those amounts change when protein content is calculated per serving. Second, protein quality, measured using the Protein Digestibility Corrected Amino Acid Score (PDCAAS) [8,9], can range from 0.52 for nuts to 0.91 for soy and 1.0 for eggs and dairy [7,8]. The 1-oz protein equivalents will change once protein content of plant foods is corrected for PDCAAS. Third, animal and plant foods in the protein foods group can differ in micronutrient content and bioavailability. Priority micronutrients from dairy include calcium, potassium, riboflavin, and vitamins A, and D [10]. Plant-based proteins in the protein foods group provide fiber, unsaturated fats, magnesium, and vitamin E [4].

The MyPlate 1-oz protein equivalency tables do not always include dairy. The Dietary Guidelines and the MyPlate protein equivalency tables have not yet considered PDCAAS. The question of affordability also needs to be raised. Milk and dairy foods are a source of affordable protein and affordable priority micronutrients [11]. Eggs and milk are consistently found to be among the least expensive protein sources [11,12]. In past analyses, eggs and milk were among the lowest-cost sources of dietary calcium, vitamin A, riboflavin, and vitamin B12 [11].

This perspective makes the argument that milk and dairy are significant sources of high-quality dietary protein for both children and adults [12] and ought to be recognized as such. By merging USDA nutrient composition data with national food prices, this study compared protein content and quality, overall nutrient density, and the affordability of milk, yogurt, and cheese with foods in the protein foods group. Nutrient density calculations were based on the well-established Nutrient Rich Food (NRF9.3) nutrient density score [[13], [14], [15], [16], [17]], calculated per 100 kcal and per serving [18]. In a novel adaptation, the protein component of the NRF9.3 score was adjusted for protein quality, using PDCAAS.

## Methods

### Nutrient composition databases

The USDA Food and Nutrient Database for Dietary Studies (FNDDS) [19,20] lists energy content and nutrient composition of foods reported as consumed by participants in the nationally representative National Health and Nutrition Examination Survey (NHANES). Individual food items in the FNDDS 2017–2018 (identified by 8-digit codes) were aggregated into food groups, food categories, and food subcategories [3,19]. The protein foods group included red meat (beef, pork, lamb, and game), poultry, seafood, eggs, beans and legumes, and nuts and seeds. The red meat category was disaggregated into subcategories of beef, pork, lamb, cured meats, and organ meats (liver). Poultry was separated into chicken and turkey. Plant proteins were separated into beans and legumes, nuts, seeds, and soy products.

The dairy group categories were milk, yogurt, and cheese [3]. Milk was disaggregated into nonfat milk, low-fat milk, reduced-fat milk and whole milk, either plain or flavored. Yogurts were separated into regular yogurt and Greek yogurt. Cheese was separated into cottage/ricotta cheese, reduced-fat cheese and hard cheese, all following USDA coding schemes.

Excluded were plant-based milks (almond, cashew) not belonging to the dairy or to the protein foods group, regular and frozen dairy desserts, egg substitutes, powdered nondairy creamers, and beans with meat, coded as mixed dishes by the USDA. The database had limited listings for tofu and none for tempeh. The final analytical database had 1514 protein and dairy items.

### Protein Digestibility Corrected Amino Acid Scores (PDCAAS)

The US Food and Drug Administration (FDA) uses PDCAAS to evaluate protein quality [8,9,21]. PDCAAS values for foods in the FNDDS 2017–2018 database were collected from a variety of published sources [[21], [22], [23], [24]] and are summarized in Supplemental Table 1. The FDA requires PDCAAS adjustment for products marketed to infants and children under 4-y old and for those products that make a protein content claim [25]. Federal regulations (21 CFR 101.9(c) [26]) specify that the corrected amount of protein per serving should be calculated by multiplying the actual protein content by its PDCAAS value [27]. This corrected protein value is then used to determine the percent daily value (%DV) for protein on the Nutrition Facts panel.

The present analyses used PDCAAS-corrected protein percent daily values (%DV) per reference amount to calculate NRF9.3 nutrient density score. Although another metric of protein quality, the Digestible Indispensible Amino Acid Score (DIAAS) [22,28], is currently preferred, DIAAS values are not available for protein foods in the FNDDS 2017–2018 database.

### Nutrient Rich Foods (NRF) Index

The NRF Index, a nutrient profiling model, is based on 2 subscores: NRn based on a variable number of nutrients to encourage and LIM based on the same 3 nutrients to limit [14,17]. The NR9 subscore was accordingly based on 9 nutrients to encourage, namely protein, fiber, calcium, iron, potassium, magnesium, vitamin A, vitamin D, and vitamin E. Protein amounts were corrected for quality by multiplying the listed protein content for each food by its PDCAAS value. The negative LIM subscore was based on the sum of percent maximum recommended values (%MRV) for 3 nutrients to limit: saturated fat, added sugar, and sodium. Standards for DV and MRV amounts came from FDA documents for back-of-pack labeling (21 CFR § 101.9) [26] and were based on 2000 kcal/d diet. The nutrient standards were protein 50 g; fiber 28 g; calcium 1300 mg; iron 18 mg; potassium 3500 mg; magnesium 420 mg, vitamin A 900 retinol activity equivalents; vitamin D 20 mcg, vitamin E 15 mg. The MRV values were saturated fat 20 g; added sugar 50 g; sodium 2300 mg.

The final NRF9.3 score was the sum of %DV for 9 nutrients to encourage minus the sum of %DV for 3 nutrients to limit. All %DV and %MRV were capped at 100%. The final NRF9.3 score is given by: NRF9.3 = NR9 – LIM.

The present analyses calculated nutrient density per 100 kcal (the standard approach) and per serving size, defined by the FDA as reference amount customarily consumed or RACC. RACC values are supposed to represent the amount of food typically consumed in 1 eating occasion and are used to define serving sizes listed on Nutrition Facts labels. For example, RACC values are 30 g for most cheeses, 170 g for yogurt, and 240 mL for milk.

### National food prices

National retail food prices for 995 dairy and protein foods came from the recently released USDA data files [29]. The USDA Purchase to Plate Price Tool collects scanner data from retailers and converts retail prices to unit prices (US$ per 100 g edible portion) [30]. Prices for 2017–2018 (released in 2023) are the most recent available. Given the complexities of food inflation, no attempt was made to adjust to post-COVID 2023 or 2024 prices.

### Affordable Nutrient Density Index

Food prices data are typically collected and expressed per 100 g of food, edible portion. The present analyses converted food prices to prices per 100 kcal of food. The Affordable Nutrient Density Index [31] is one metric to assess the amount of calories or nutrients per penny. To be nutrition-relevant, economic indicators need to be expressed in terms of monetary cost per calorie or per nutrient, as opposed to food weight [32].

The Affordable Nutrient Density Index, a ratio of food nutrient density to food price, helps to identify those foods in the protein foods group that provide most protein or other nutrients in relation to food price [10,12,17,32]. Calculations can be conducted per 100 kcal or per serving. Also calculated was the monetary cost of 7 g of high-quality protein, the basis of 1-ounce protein equivalents in the Dietary Guidelines for Americans (DGA).

### USDA 1-ounce protein equivalents

The term “1-ounce equivalent of protein” refers to the amount of food that provides roughly 7 g of protein in the context of a balanced diet. The USDA MyPlate [2] provides a chart of what foods provide 1-ounce protein equivalents. These data, summarized in Table 1, were intended to help standardize protein intake from various food groups, using easy to understand household measures. In the present analyses, protein equivalents in ounces, cups, and tablespoons were converted to grams, and were used to calculate 1-oz protein equivalents for each food category in the FNDDS 2017–2018 database.

**TABLE 1:** Amounts of foods in the protein foods group that count as 1-oz protein equivalents.

	Amount that counts as 1-oz equiv in the protein foods group
Meats	1 ounce cooked lean beef, pork, or lamb (incl. ground) 1 ounce cooked game meats (deer, venison, bear, rabbit) 1 slice of luncheon or deli meats (beef, chicken, ham, pork, turkey) 1 ounce cooked organ meats
Poultry	1 ounce cooked (without skin) chicken, ostrich, or turkey 2 ounces cooked Cornish hen, duck, goose, pheasant, or quail
Seafood	1 ounce cooked finfish (catfish, cod, flounder, freshwater trout, haddock, hake, halibut, herring, light tuna, mackerel, mullet, perch, pollock, salmon, sea bass, snapper, sole, tilapia, whiting) 1 ounce cooked shellfish (clams, crab, crayfish, lobster, mussels, octopus, oysters, scallops, shrimp, squid) 1 ounce canned fish (anchovies, trout, herring, tuna, salmon, sardines)
Eggs	1 egg
Nuts, seeds, soy products	½ ounce of nuts (almonds, pistachios, walnuts) ½ ounce of seeds (sunflower, chia, flax, pumpkin, sesame), roasted 1 tablespoon (16 g) of almond, cashew, or peanut butter ¼ cup (∼2 ounces) of tofu
Beans, peas, lentils	¼ cup of cooked beans, peas or lentils (black, fava, kidney, lima, mung, navy, pink, pinto, or soy), white beans, black-eyed peas (cow peas), chickpeas, lentils, baked beans or refried beans

Amounts are for food categories, subcategories, and individual item.

The FNDDS 2017–2018 database was recoded and trimmed to include cooked beef, pork and lamb, chicken and turkey without skin, game meats, including bear and elk, and game birds. Cured meats (luncheon and deli) and liver and organ meats were included, as were eggs and omelets. All the cooked fish and shellfish listed, including fried, were included. Nuts and nut mixtures (other than trail mix with chocolate) were included and so were nut butters. All the cooked beans, peas, and lentils in the database were included. Excluded were giblets, neck bones, egg salads with mayonnaise, mixed egg dishes with added vegetables, and bean dishes with meat. Raw fish and shellfish and any fish not listed (barracuda, shark, eel) were excluded. The goal was to compare milk and dairy with nutrient-dense forms of foods in the protein foods groups, preferably uncomplicated by the addition of fat, saturated fat, starches, or salt. The final analytical data base of 947 food items was used to calculate MyPlate 1-oz protein equivalents.

The DGA recommended amounts were not always consistent. For example, the DGA clearly lists ½ oz of nuts (14 g) and 1 tbs (16 g) of nut butter, including peanut butter [2]. Even though other sources [33] recommend 1 oz of nuts (28 g) and 2 tbs (32 g) of nut butter, the present decision was to follow the USDA MyPlate standards exactly [2].

### Plan of analysis

Relevant differences between means for protein amount and quality, nutrient density scores, and estimated cost per reference amount were tested using 1-way analysis of variance (ANOVA) by What We Eat in America food group or category.

## Results

### Protein content of foods per 100 g and per serving

**FIGURE 1:**
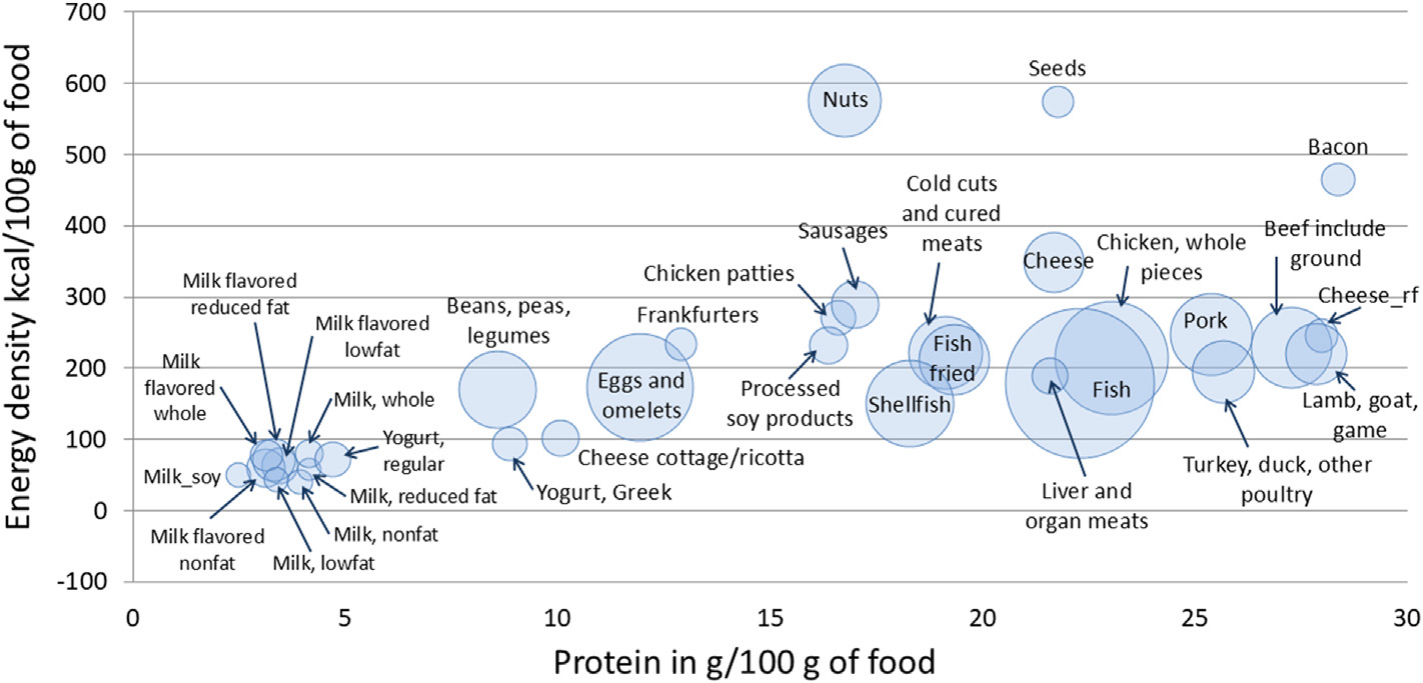
A scatterplot of energy density (kcal/100 g) and protein content in grams per 100 g of food for dairy and protein foods by food category. The size of the bubble reflects the number of items in each category in the Food and Nutrient Database for Dietary Studies (FNDDS) 2017–2018.

Analyses were conducted with a database of 1514 foods in the dairy and protein food groups. Figure 1 shows mean protein content in grams of protein per 100 g of food, by food category, plotted against mean energy density (kcal/100 g). The size of the bubble reflects the number of foods in each category. Meat and poultry had the highest mean protein content per 100 g of food. Pork items contained a mean of 25.4 g of protein per 100 g, as compared with 27.3 g/100 g for beef and 23.2 g/100 g for chicken. Nuts contained a mean of 16.8 g protein per 100 g and seeds contained 21.8 g/100 g. Mean protein content of the beans, peas, and legumes category was 8.6 g/100 g of protein.

Milks and flavored milks provided 3–4 g of protein per 100 g product. Mean protein content was highest for Greek yogurt (8.9 g/100 g), cottage cheese (10.1 g/100 g), and regular cheese (21.7 g/100 g). Those values were comparable with eggs and omelets and with pulses.

### Protein Digestibility Corrected Amino Acid Score (PDCAAS)

**FIGURE 2:**
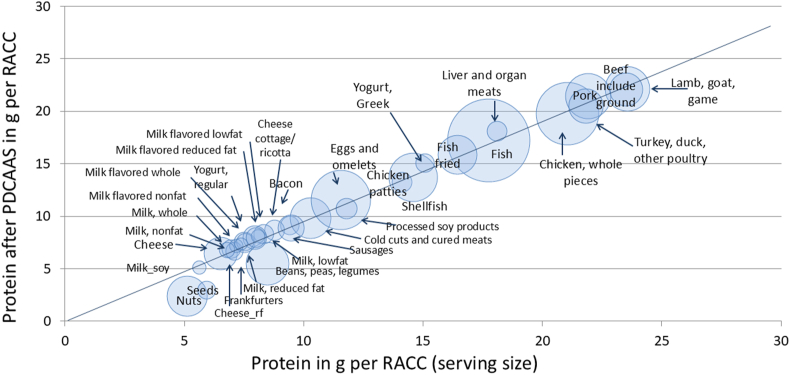
A scatterplot of protein content per serving (RACC) of food plotted against protein content in grams per serving corrected using Protein Digestibility Corrected Amino Acid Score (PDCAAS) by food category. The size of the bubble reflects the number of items in each category in the Food and Nutrient Database for Dietary Studies (FNDDS) 2017–2018. RACC, Reference Amounts Customarily Consumed.

Figure 2 shows the amounts of protein in grams per serving (g/RACC) plotted against amounts of protein per serving following PDCAAS correction. First, expressing protein in amounts per serving minimized any differences in protein content between dairy products and some foods in the protein foods group. When calculated per serving, the amount of protein in milk (245 g) or yogurt (170 g) was still lower than in meat (85 g) but was now in the same range as eggs and omelets, pulses, legumes, and nuts and seeds. Second, unlike plant proteins (except soy), milk and dairy were not affected by the PDCAAS correction.

### Protein affordability metrics

Protein is a desirable but costly nutrient. Higher protein content per 100 g was associated with higher food prices per 100 g (Pearson correlation coefficient 0.484). The highest prices per 100 g of food were for shellfish, lamb, fish, and beef, consistent with past findings [34]. Mean national prices for pork were closer to chicken and turkey [7]. Milk, yogurt, and cottage cheese had some of the lower mean prices per 100 g along with eggs, pulses, and legumes**.** Mean prices for milk and dairy per serving were in the same range as eggs, beans, peas and legumes, and nuts and seeds. Calculation of prices per 50 g of protein (equivalent to 100% DV) showed that the cost of protein was lowest for nonfat milk ($1.62) and whole milk ($1.96), followed by pork ($2.00), chicken ($2.11), eggs and omelets ($2.20), and beans, peas and legumes ($2.22). The highest prices for 50 g of protein were for shellfish ($9.23), nuts ($6.72), and bacon ($5.38). These data are presented in Supplemental Table 2.

### NRF9.3 nutrient density scores

**FIGURE 3:**
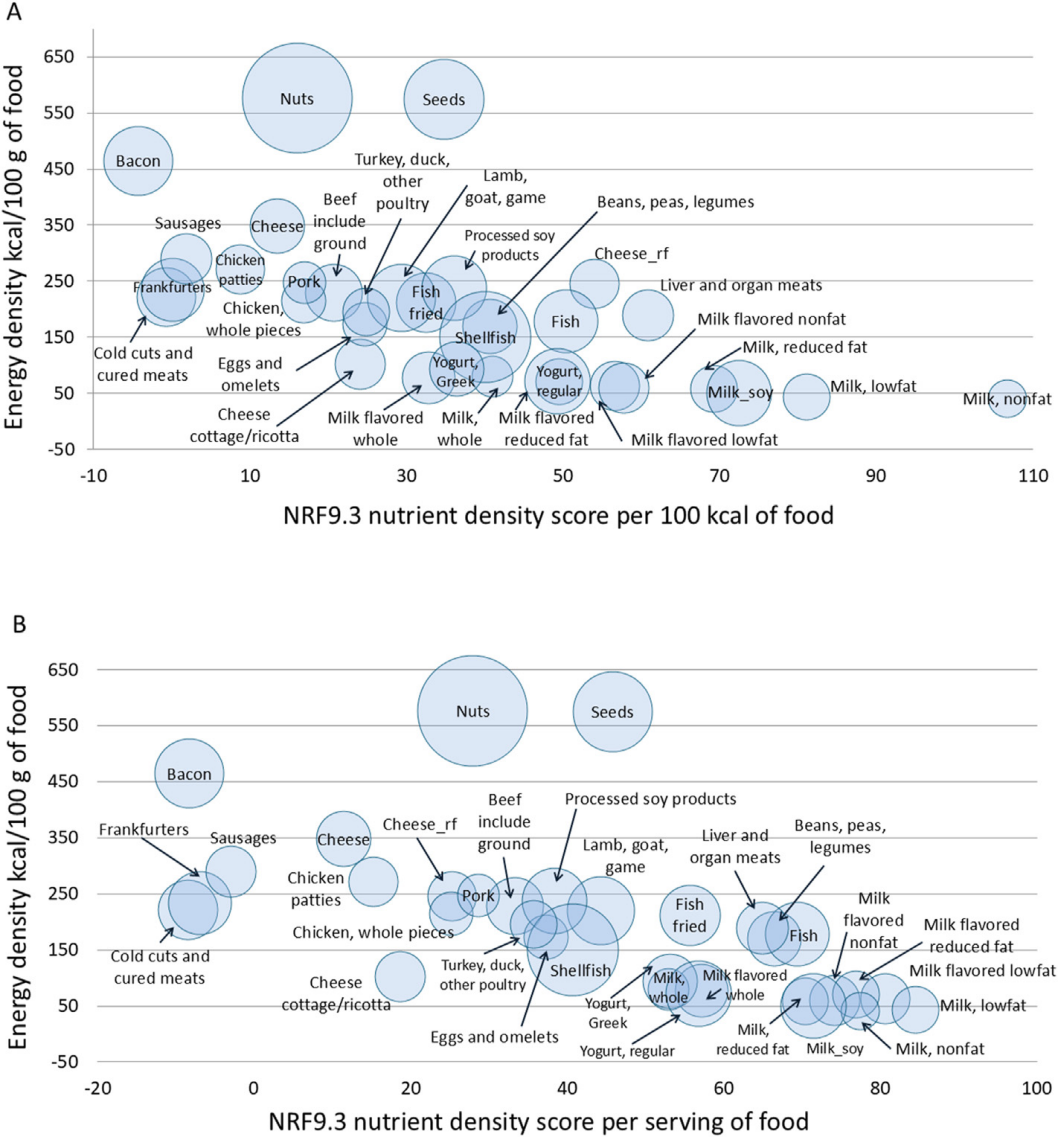
A scatterplot of mean Nutrient Rich Food Index (NRF9.3) nutrient density scores per 100 kcal (A) and per serving (RACC) (B) plotted against mean energy density (kcal/100 g) of foods by food category. The size of the bubble reflects the price per 100 kcal (A) and per serving (B) from the USDA national food prices database. RACC, Reference Amounts Customarily Consumed.

Figure 3A shows mean NRF9.3 nutrient density values calculated per 100 kcal and plotted against energy density (kcal/100 g). Amounts of protein were corrected for PDCAAS. The size of the bubble reflects mean food prices per 100 kcal of food. Reduced-fat and low-fat milk, as well as soy milk, had low energy density and received high nutrient density scores. Low-fat milks were followed by fish and seafood, pulses, whole milk, and regular and Greek yogurts. Consistent with other calculations, cheese got a low score largely because of sodium and saturated fat. As can be seen from the sizes of the bubbles, food prices per 100 kcal were lower for dairy than for meat or fish.

Figure 3B shows mean NRF9.3 nutrient density values calculated per serving and plotted against energy density (kcal/100 g). Amounts of protein were corrected for PDCAAS. The size of the bubble reflects mean food prices per serving of food. The highest mean nutrient density scores were for skim milk, low-fat milk, and reduced-fat milk, followed by canned fish, organ meats, and pulses, consistent with past reports. Food prices per serving were lower for dairy than for meat and fish. These prices are provided in Supplemental Table 3.

### Protein equivalents in the DGA

Table 2 shows that the USDA values for 1-oz protein equivalents were 1 oz (28 g) for meat, poultry, fish and seafood; 1 egg (60 g); ¼ cup of cooked beans (60 g); 1/2 or 1 oz (28 g) of nuts; 1 or 2 tbs of peanut butter (32 g); and ¼ cup of tofu. Those values have not been corrected for PDCAAS. Also shown in Table 2 are the calculated mean (and median) amounts of food, in grams and by category, that would provide 7 g of protein after PDCAAS correction. For meat and poultry, the amounts were very close to 1 oz. For fish and seafood, the amounts were in the 1.0–1.5 oz range, because cooked fish in the FNDDS database were often breaded and fried. The amounts of game birds, shellfish, and deli and luncheon meats that provided 7 g of protein were in the 1.0–1.5 oz range. The amount of eggs (1 egg) was an exact match.

**TABLE 2:** USDA 1-oz protein equivalents, foods amounts needed to provide 7 g of protein and protein cost (PDCAAS corrected).

	USDA 1-oz eq	Amount (g) of food providing 7 g protein		Recalculated 1-oz equivalents	Price per 7 g protein ($)	
		Mean/SEM	Median		Mean/SEM	Median
Meat						
Beef	1-oz (28 g)	27.0 ± 0.4	25.7		0.50 ± 0.02	0.47
Pork	1-oz (28 g)	28.9 ± 0.6	28.0		0.25 ± 0.00	0.26
Lamb	1-oz (28 g)	30.0 ± 0.7	30.0		0.85 ± 0.03	0.83
Game meats	1-oz (28 g)	25.4 ± 0.6	25.5		0.59 ± 0.03	0.53
Organ meats	1-oz (28 g)	36.0 ± 3.4	31.1		0.43 ± 0.14	0.25
Deli meats	1-oz (28 g)	43.6 ± 1.7	40.5	1.0–1.5-oz (42g) g)	0.53 ± 0.04	0.50
Poultry						
Chicken	1-oz (28 g)	30.9 ± 0.5	30.9		0.30 ± 0.02	0.27
Turkey	1-oz (28 g)	27.0 ± 0.5	26.6		0.33 ± 0.03	0.28
Game birds	2-oz (56 g)	30.5 ± 1.0	31.9	1-oz (28 g)	0.59 ± 0.00	0.59
Seafood						
Finfish	1-oz (28 g)	35.5 ± 0.7	34.8		0.66 ± 0.03	0.63
Fish, fried	1-oz (28 g)	38.9 ± 0.9	38.7		0.58 ± 0.06	0.58
Fish, canned	1-oz (28 g)	31.9 ± 1.7	33.6		0.49 ± 0.10	0.45
Shellfish	1-oz (28 g)	48.1 ± 2.4	43.2	1.5-oz (42 g)	1.42 ± 0.12	1.35
Eggs	1 egg (60 g)	59.6 ± 1.0	59.7	1 egg (60 g)	0.28 ± 0.02	0.24
Nuts, seeds, soy						
Nuts	1/2 oz (14 g) 1-oz (28 g)	94.9 ± 5.7	76.0	3-oz (84 g)	1.98 ± 0.26	1.45
Peanut butter	1 tbs (16 g); 2 tbs (32 g)	70.8 ± 6.7	66.2	4 tbs (64 g)	0.77 ± 0.32	0.34
Seeds	½ oz (14 g)	66.9 ± 3.7	72.4	2.5 oz (70g)	1.05 ± 0.20	0.80
Tofu	¼ cup (56 g)	53.9 ± 6.5	40.9	¼ cup (56 g)	0.69 ± 0.13	0.55
Beans, pulses	¼ cup (60 g)	145.1 ± 5.2	138	½ cup (120 g)	0.46 ± 0.02	0.42
Dairy group						
Cheese		31.7 ± 1.0	30.2	1 oz (28 g)	0.47 ± 0.04	0.40
Cheese lower fat		26.4 ± 1.6	25.1	1 oz (28 g)	0.39 ± 0.05	0.34
Cheese, cottage		63.1 ± 3.0	64.3	¼ cup (56 g)	0.38 ± 0.07	0.31
Yogurt, Greek		79.6 ± 2.1	81.0	½ cup (85 g)	0.48 ± 0.02	0.43
Yogurt, regular		155 ± 8.8	138	1 cup (170 g)	0.71 ± 0.04	0.67
Milk, whole		210 ± 7.3	213	1 cup (245 g)	0.28 ± 0.08	0.30
Milk, reduced fat		201 ± 7.7	209	1 cup (245 g)	0.35 ± 0.08	0.43
Milk, low fat		210 ± 2.2	207	1 cup (245 g)	0.27 ± 0.08	0.28
Milk, skim		210 ± 2.8	209	1 cup (245 g)	0.21 ± 0.08	0.14
Milk, soy		333.9 ± 11.8	332	1 cup (245 g)	0.47 ± 0.03	0.45

Abbreviation: PDCAAS, Protein Digestibility Corrected Amino Acid Score.

For some foods, the listed “equivalency” amounts did not provide anywhere near 7 g of protein. For example, the mean amount of cooked pulses was 145 g. That amount was closer to ½ cup (120 g) than to the suggested ¼ cup of pulses. The recalculated gram amounts were converted into 4 tbs for peanut butter rather than the suggested 2 tbs, 2.5 oz for seeds rather than the suggested ½ oz, and 3 oz of nuts rather than the suggested 1 oz. The amount of tofu was the same (1/4 cup) in the 1-oz equivalence tables and in the recalculated values. Because soy milks were listed as having <3 g of protein per 100 g, the amount needed to provide 7 g of protein was 10 oz of soy milk.

By contrast, 7 g of protein was provided by 1 oz (28–33 g) of cheese and reduced-fat cheese, by >¼ cup for cottage cheese (75 g), ∼½ cup for Greek yogurt (80 g), and by <1 serving of yogurt (155 g) and fluid milk (range 183–208 g). Those values can be used to establish 1-oz protein equivalents for milk and dairy, namely 1 oz of cheese (28 g), ¼ cup (56 g) of cottage cheese; ¾ cup of Greek yogurt (170 g); and 1 cup (245 g) of regular yogurt and fluid milk. Those values roughly correspond to RACC values used by the FDA. Also shown in Table 2 are prices for 7 g of protein corrected for PDCAAS.

## Discussion

This perspective makes the argument that dairy foods are nutrient-rich, provide affordable high-quality protein, and compare favorably with many foods in the USDA protein foods group. The present analyses, based on USDA nutrient composition data and on national food prices, showed that low-fat milk, yogurt, and cottage cheese received high NRF9.3 nutrient density scores when calculated per 100 kcal and per serving. Importantly, mean prices per 50 g of protein (equivalent to 100% DV) were lower for low-fat milk than for pork, chicken, eggs and omelets, and beans, peas, and legumes.

Milk and dairy have less protein per 100 g than do meat, poultry, and fish. However, those differences were reduced when protein amounts were calculated per serving. The differences between dairy and plant proteins in the protein foods group were reduced further after adjustments for protein quality using PDCAAS. Greek yogurt and cottage cheese provided the necessary 1-ounce equivalent of protein (7 g) per serving that was also provided by less than a serving (8 oz or 245 g) of milk.

The equivalence of protein sources has been questioned before [[34], [35], [36]]. The FDA requires that PDCAAS correction be applied to foods that make a protein content claim [25,27]. Adjusting the protein content of animal- and plant-based foods in the protein food group suggests that the protein equivalency chart may need some corrections [2]. First, meat and poultry categories did provide a mean of 7 g of protein per ounce (28–30 g). The same amount was contributed by 60 g of eggs (1 egg) and by 2 ounces of tofu.

Dairy products that provided 7 g of protein were 1 serving of cheese (30 g) or cottage cheese (76 g), under ½ serving of Greek yogurt (80 g), under 1 serving of yogurt (160 g) and 6 oz (180 g) of whole, reduced-fat or skim milk. By contrast, the USDA equivalency amounts of nuts (1 oz), peanut butter (32 g), and beans, peas, and legumes did not come close to providing the 7 g of protein, either before or after the PDCAAS correction. The necessary median values were provisionally estimated (given the limitations of the FNDDS database) at 120 g for nuts, 78 g for peanut butter, and 143 g for beans. The present results are consistent with studies showing that dairy foods in the United States diet are difficult to replace [6,37,38].

Most studies agree that protein, and especially animal protein, is the nutrient with the highest monetary and environmental costs. Assuring access to affordable high-quality protein is a major priority for public health nutrition and one of the pillars of the USDA Thrifty Food Plan. The present calculations have estimated the cost of obtaining 50 g of high-quality protein from different food categories in the dairy and protein food groups. Milk and dairy, along with eggs and the beans, peas, and lentils category, were among the lowest-cost protein foods.

The present calculations of nutrient density, based on the well-established NRF9.3 nutrient density score, used protein content corrected for PDCAAS. Nutrient density scores for milk and dairy were very favorable for milk (nonfat, low fat, reduced-fat, and whole milk) and for yogurt and cottage cheese. Ratings were lower for cheese because of high content of sodium and saturated fat. The PDCAAS correction is most valuable when people (e.g., infants or children) derive most of their protein from a particular food. It is less critical for adults who consume a wider variety of foods so that any amino acid imbalance can be corrected by amino acids from another source. However, as long as the MyPlate protein equivalency tables are making an implicit protein content claim, they need to be corrected for PDCAAS in line with FDA guidelines. The present calculations may need to be modified once the DIAAS data become more available.

The use of FNDDS data with foods as consumed was both an asset and a limitation. The FNDDS database provides a direct link to diets of NHANES participants and their health outcomes, something that the SR28 Legacy does not do. However, those foods are not always the most nutrient-dense versions and tend to be cooked in a variety of ways. For example, many people may get their protein by eating fried chicken or fried fish; those foods—as consumed—need to be included in calculations of nutrient density or protein equivalents.

### Future dietary guidelines

The United States Department of Health and Human Services and USDA are in receipt of proposals to shift emphasis toward plant-based proteins in the 2025 edition of DGA. The proposal from the Dietary Guidelines Advisory Committee was to restructure the protein foods group, giving priority to nuts, seeds, and soy over the traditional meat, eggs, poultry, and fish [39]. Another proposal was to reclassify pulses as key protein sources instead of vegetables [39]. The 1-oz protein equivalency tables assume an additional importance in this context, showing that lager amounts of plant-based foods are needed to provide the requisite 7 g of high-quality protein. That criterion is easily met by milk and dairy. Along with pulses, dairy could be a part of the protein food group given its high nutrient content, amount of protein per serving, and relatively low cost.

## Author contributions

The sole author was responsible for all aspects of this manuscript.

## Data availability

Data described in the manuscript, code book, and analytic code will be made publicly and freely available without restriction at FoodData Central (https://fdc.nal.usda.gov/download-datasets.html).

## Funding

This work was supported by Dairy Management Inc. The funder had no involvement is study design, data analyses or interpretation, or the decision to submit this work for publication.

## Conflict of interest

AD is the original developer of the Naturally Nutrient Rich (NNR) and the Nutrient Rich Food (NRF) nutrient profiling models and is or has been a member of scientific advisory panels for BEL, Lesaffre, Nestlé, FrieslandCampina, National Pork Board, and Carbohydrate Quality Panel supported by Potatoes USA. AD has worked with Ajinomoto, Ayana Bio, DSM-Firmenich, FoodMinds, Kraft Heinz, Meiji, MS-Nutrition, Nutrition Impact LLC, Nutrition Institute, PepsiCo, Samsung, and Soremartec on quantitative ways to assess nutrient density of foods.
