# Supplementary material for: Perspective: Milk and Dairy Provide Affordable High-Quality Protein and Merit Inclusion in the Protein Foods Group
Source: Curr Dev Nutr. 2024 Dec 21;9(1):104539. doi: 10.1016/j.cdnut.2024.104539 (PMC11787005; doi:10.1016/j.cdnut.2024.104539)
Supplement: multimedia component 1 [file mmc1.docx]

SUPPLEMENTAL MATERIALS

Perspective: Milk and dairy provide affordable high-quality protein and merit inclusion in the Protein Foods Group

Adam Drewnowski ^1,^*

^1^ Center for Public Health Nutrition, University of Washington, Seattle, WA 98195-3410, USA

***** Correspondence: Adam Drewnowski adamdrew@uw.edu

Supplemental Table S1. Estimated PDCAAS values for protein food group categories

| Milk, dairy and eggs | Milk, yogurt | 1.00 |
| --- | --- | --- |
|  | Cheese | 0.99 |
| Eggs | Egg | 1.00 |
| Meat and poultry | Beef, organ meats | 0.94 |
|  | Chicken | 0.94 |
|  | Pork | 0.98 |
|  | Other meats | 0.94 |
| Fish and seafood | Tuna, sardine | 1.00 |
|  | Shellfish | 0.94 |
|  | Other fish | 0.94 |
| Soy | Soybeans | 0.91 |
| Beans, peas, lentils | Black beans | 0.61-0.70 |
|  | Brown beans | 0.61 |
|  | Kidney beans | 0.68-0.72 |
|  | White beans | 0.61 |
|  | Navy beans | 0.61 |
|  | Pinto beans | 0.63 |
|  | Red beans | 0.61 |
|  | Cowpeas | 0.38 |
|  | Lentils | 0.52-0.70 |
|  | Peas | 0.69 |
|  | Cooked peas | 0.60 |
|  | Chickpeas | 0.70 |
|  | Legumes and peas | 0.70 |
|  | Legumes | 0.74 |
| Nuts and seeds | Nuts and seeds | 0.47 (mean) |
|  | Almonds | 0.40-0.50 |
|  | Walnuts | 0.40-0.50 |
|  | Cashews | 0.50-0.60 |
|  | Pistachios | 0.70-0.75 |
|  | Peanut butter | 0.52-0.60 |

Supplemental Table S2: Protein amounts per RACC and protein cost (per 7 g and per 50g) after PDCAAS correction

| weia22_text | N | Protein g/RACC | SEM | Food Price  $/RACC | SEM | Price  7 g protein | SEM | Price  50g protein | SEM |
| --- | --- | --- | --- | --- | --- | --- | --- | --- | --- |
| Bacon | 13 | 9.14 | 2.06 | 1.00 | 0.25 | 0.78 | 0.12 | 5.39 | 10 |
| Beans, peas, legumes | 74 | 5.52 | 0.24 | 0.37 | 0.04 | 0.48 | 0.03 | 2.22 | 55 |
| Beef include ground | 81 | 22.14 | 0.34 | 1.66 | 0.10 | 0.53 | 0.03 | 3.56 | 42 |
| Cheese | 44 | 6.46 | 0.26 | 0.44 | 0.03 | 0.49 | 0.03 | 3.44 | 38 |
| Cheese cottage/ricotta | 16 | 8.69 | 0.76 | 0.51 | 0.05 | 0.41 | 0.06 | 2.92 | 12 |
| Cheese_reduced fat | 13 | 6.89 | 0.70 | 0.40 | 0.06 | 0.39 | 0.05 | 2.79 | 13 |
| Chicken patties | 15 | 13.27 | 0.49 | 0.73 | 0.03 | 0.39 | 0.02 | 2.61 | 15 |
| Chicken, whole pieces | 161 | 19.78 | 0.29 | 0.87 | 0.04 | 0.31 | 0.01 | 2.11 | 131 |
| Cold cuts and cured meats | 67 | 9.83 | 0.34 | 0.76 | 0.05 | 0.57 | 0.03 | 3.86 | 46 |
| Eggs and omelets | 142 | 11.55 | 0.31 | 0.51 | 0.02 | 0.31 | 0.01 | 2.20 | 97 |
| Fish | 279 | 17.23 | 0.21 | 1.60 | 0.07 | 0.66 | 0.03 | 4.58 | 118 |
| Fish fried | 61 | 15.88 | 0.30 | 1.27 | 0.13 | 0.59 | 0.06 | 4.02 | 20 |
| Frankfurters | 12 | 6.67 | 0.34 | 0.59 | 0.13 | 0.65 | 0.14 | 4.34 | 9 |
| Lamb, goat, game | 45 | 22.09 | 0.50 | 2.28 | 0.07 | 0.75 | 0.03 | 5.05 | 29 |
| Liver and organ meats | 15 | 18.09 | 1.54 | 0.84 | 0.16 | 0.43 | 0.14 | 3.06 | 11 |
| Milk flavored lowfat | 15 | 8.31 | 0.20 | 0.47 | 0.05 | 0.40 | 0.05 | 2.87 | 15 |
| Milk flavored nonfat | 17 | 7.51 | 0.50 | 0.40 | 0.04 | 0.41 | 0.05 | 2.90 | 16 |
| Milk flavored reduced fat | 22 | 7.95 | 0.19 | 0.40 | 0.03 | 0.35 | 0.02 | 2.51 | 21 |
| Milk flavored whole | 12 | 7.55 | 0.60 | 0.39 | 0.02 | 0.45 | 0.11 | 3.22 | 12 |
| Milk, lowfat | 7 | 8.09 | 0.11 | 0.41 | 0.12 | 0.35 | 0.10 | 2.49 | 5 |
| Milk, nonfat | 7 | 7.17 | 0.82 | 0.21 | 0.08 | 0.23 | 0.07 | 1.62 | 5 |
| Milk, reduced fat | 6 | 7.37 | 1.07 | 0.36 | 0.12 | 0.35 | 0.06 | 2.48 | 4 |
| Milk, whole | 9 | 6.78 | 0.92 | 0.24 | 0.07 | 0.27 | 0.05 | 1.96 | 6 |
| Milk_soy | 7 | 5.11 | 0.16 | 0.51 | 0.18 | 0.69 | 0.22 | 4.49 | 7 |
| Nuts | 66 | 2.41 | 0.11 | 0.51 | 0.04 | 2.00 | 0.23 | 6.72 | 61 |
| Pork | 83 | 21.47 | 0.57 | 0.89 | 0.04 | 0.29 | 0.02 | 2.00 | 53 |
| Processed soy products | 17 | 9.80 | 1.34 | 0.75 | 0.10 | 0.69 | 0.13 | 4.49 | 11 |
| Sausages | 27 | 8.90 | 0.35 | 0.53 | 0.04 | 0.42 | 0.05 | 2.85 | 24 |
| Seeds | 12 | 2.97 | 0.33 | 0.38 | 0.05 | 1.05 | 0.20 | 3.75 | 12 |
| Shellfish | 94 | 13.71 | 0.45 | 2.63 | 0.19 | 1.37 | 0.10 | 9.23 | 43 |
| Turkey, duck, other poultry | 48 | 20.51 | 0.48 | 1.07 | 0.07 | 0.36 | 0.03 | 2.42 | 26 |
| Yogurt, Greek | 14 | 15.08 | 0.40 | 1.01 | 0.02 | 0.48 | 0.02 | 3.40 | 14 |
| Yogurt, regular | 15 | 7.99 | 0.38 | 0.86 | 0.08 | 0.71 | 0.04 | 5.08 | 15 |

Supplemental Table S3: Energy density, protein cotents (per 100g and per serving) and NRF9.3 nutrient density scores calculated per 100 kcal of food and per serving. RACC: Reference Amounts Customarity Consumed.

| wweia22_text | N | kcal/100g | SEM | Protein g/100g | SEM | Protein g/RACC | SEM | NRF9.3 100 kcal | NRF9.3 RACC |
| --- | --- | --- | --- | --- | --- | --- | --- | --- | --- |
| Bacon | 13 | 465 | 36 | 28.38 | 3.08 | 9.14 | 2.06 | -4.29 | -8.3 |
| Beans, peas, legumes | 74 | 171 | 7 | 8.65 | 0.47 | 5.52 | 0.24 | 40.62 | 66.47 |
| Beef include ground | 81 | 229 | 6 | 27.27 | 0.45 | 22.14 | 0.34 | 20.74 | 33.29 |
| Cheese | 44 | 348 | 8 | 21.69 | 0.68 | 6.46 | 0.26 | 13.49 | 11.48 |
| Cheese cottage/ricotta | 16 | 102 | 14 | 10.07 | 0.72 | 8.69 | 0.76 | 24.03 | 18.64 |
| Cheese_rf | 13 | 246 | 26 | 27.98 | 1.62 | 6.89 | 0.70 | 53.95 | 25.29 |
| Chicken patties | 15 | 271 | 10 | 16.61 | 0.61 | 13.27 | 0.49 | 8.75 | 15.28 |
| Chicken, whole pieces | 161 | 215 | 4 | 23.05 | 0.28 | 19.78 | 0.29 | 16.86 | 25.22 |
| Cold cuts& cured meats | 67 | 222 | 12 | 19.14 | 0.74 | 9.83 | 0.34 | -0.66 | -8.4 |
| Eggs and omelets | 142 | 173 | 5 | 11.95 | 0.18 | 11.55 | 0.31 | 24.69 | 37.31 |
| Fish | 279 | 179 | 3 | 22.29 | 0.43 | 17.23 | 0.21 | 50.32 | 69.37 |
| Fish fried | 61 | 212 | 4 | 19.33 | 0.34 | 15.88 | 0.30 | 32.47 | 55.69 |
| Frankfurters | 12 | 234 | 25 | 12.90 | 0.67 | 6.67 | 0.34 | 0.16 | -6.85 |
| Lamb, goat, game | 45 | 220 | 9 | 27.86 | 0.59 | 22.09 | 0.50 | 29.33 | 44.31 |
| Liver and organ meats | 15 | 189 | 15 | 21.60 | 1.68 | 18.09 | 1.54 | 60.83 | 64.94 |
| Milk flavored lowfat | 15 | 63 | 3 | 3.46 | 0.09 | 8.31 | 0.20 | 56.61 | 80.63 |
| Milk flavored nonfat | 17 | 60 | 4 | 3.13 | 0.21 | 7.51 | 0.50 | 57.75 | 74.17 |
| Milk flavored reduced fat | 22 | 71 | 3 | 3.31 | 0.08 | 7.95 | 0.19 | 49.47 | 76.88 |
| Milk flavored whole | 12 | 78 | 4 | 3.14 | 0.25 | 7.55 | 0.60 | 32.81 | 57.21 |
| Milk, lowfat | 7 | 43 | 2 | 3.37 | 0.05 | 8.09 | 0.11 | 81.08 | 84.46 |
| Milk, nonfat | 7 | 41 | 6 | 3.93 | 0.60 | 7.17 | 0.82 | 106.69 | 77.43 |
| Milk, reduced fat | 6 | 58 | 7 | 4.15 | 0.67 | 7.37 | 1.07 | 69.27 | 70.41 |
| Milk, whole | 9 | 80 | 10 | 4.15 | 0.51 | 6.78 | 0.92 | 40.95 | 52.94 |
| Milk_soy | 7 | 50 | 9 | 2.49 | 0.18 | 5.11 | 0.16 | 72.48 | 71.46 |
| Nuts | 66 | 576 | 10 | 16.76 | 0.77 | 2.41 | 0.11 | 16.01 | 27.94 |
| Pork | 83 | 248 | 9 | 25.39 | 0.64 | 21.47 | 0.57 | 16.94 | 28.66 |
| Processed soy products | 17 | 232 | 30 | 16.38 | 1.40 | 9.80 | 1.34 | 36.10 | 38.36 |
| Sausages | 27 | 290 | 10 | 17.01 | 0.72 | 8.90 | 0.35 | 1.83 | -2.94 |
| Seeds | 12 | 575 | 7 | 21.78 | 1.43 | 2.97 | 0.33 | 34.74 | 45.83 |
| Shellfish | 94 | 150 | 6 | 18.29 | 0.89 | 13.71 | 0.45 | 40.06 | 40.77 |
| Turkey, duck, poultry | 48 | 195 | 7 | 25.67 | 0.60 | 20.51 | 0.48 | 24.85 | 35.73 |
| Yogurt, Greek | 14 | 94 | 7 | 8.87 | 0.23 | 15.08 | 0.40 | 36.35 | 53.17 |
| Yogurt, regular | 15 | 73 | 3 | 4.61 | 0.23 | 7.99 | 0.38 | 49.25 | 56.79 |
